# Supplementary material for: Protective and risk factors of mental health of working age adults with adventitious total bilateral blindness and low vision: A scoping review protocol
Source: PLoS One. 2024 Jan 10;19(1):e0296659. doi: 10.1371/journal.pone.0296659 (PMC10781084; doi:10.1371/journal.pone.0296659)
Supplement: S1 Appendix — (DOCX) [file pone.0296659.s001.docx]

# S1 Appendix : Full search strategy

# Medline (Ovid)

Preliminary Search was carried out on the 28^th^ of March 2023 and subsequently refined.

| **Components** | **Search Terms** | **Records** |
| --- | --- | --- |
| Blindness and Low Vision  (Combined with Boolean term “OR”) | 1. exp Blindness/  2. Blindness.tw.  3. (visual$ adj1 impair$).tw.  4. (vision adj1 impair$).tw.  5. (vision adj1 loss).tw.  6. (loss adj3 vision).tw.  7. (low adj1 vision).tw.  8. (partial$ adj1 sight$).tw. | 100,771 |
| Mental Health  (Combined with Boolean term “OR”) | 9. exp Mental Health/  10. (mental adj1 health).tw.  11. (mental adj1 well-being).tw.  12. exp Depression/  13. depress$.tw.  14. exp Anxiety/  15. anxiety.tw.  16. (Post adj1 traumatic adj1 stress adj1 disorder$).tw.  17. exp Psychological Distress/  18. (Psychological adj1 distress).tw.  19. exp "Quality of Life"/  20. (quality adj3 life).tw.  21. exp Suicide/  22. suicide.tw. | 1,956,824 |
| Working Age Adults | 23. exp Adult/  24. adult$.tw.  25. exp Middle Aged/  26. (middle adj1 age$).tw.  27. (working adj1 age$ adj1 adult$).tw.  28. (18-65 adj1 year$).tw. | 14,057,388 |
| Combined with Boolean term “OR” | 29. 1 or 2 or 3 or 4 or 5 or 6 or 7 or 8 | 72,003 |
| Combined with Boolean term “OR” | 30. 9 or 10 or 11 or 12 or 13 or 14 or 15 or 16 or 17 or 18 or 19 or 20 or 21 or 22 | 1,168,355 |
| Combined with Boolean term “OR” | 31. 23 or 24 or 25 or 26 or 27 or 28 | 8,478,099 |
| Combined with Boolean term “AND” | 32. 29 and 30 and 31 | 2,532 |
| Total after limiting to English language, Human and Medline | | 2,346 |
