# Supplementary material for: Protective and risk factors of mental health of working age adults with adventitious total bilateral blindness and low vision: A scoping review protocol
Source: PLoS One. 2024 Jan 10;19(1):e0296659. doi: 10.1371/journal.pone.0296659 (PMC10781084; doi:10.1371/journal.pone.0296659)
Supplement: S2 Appendix — (DOCX) [file pone.0296659.s002.docx]

# **S2 Appendix: Data extraction template**

| **Title, First Author and Year** | **Country of Publication** | **Aims** | **Study**  **Methods** | **Age** | **Sex** | **Number**  **Of**  **People** | **Type of Vision Loss**  **(Total blindness and/or Low Vision)** | **Key findings**  **Protective Factors** | **Key Findings**  **Risk Factors** |
| --- | --- | --- | --- | --- | --- | --- | --- | --- | --- |
|  |  |  |  |  |  |  |  |  |  |

Source: Adapted from Peters et al. (2020a)
